# Poor Homologous Synapsis 1 Interacts with Chromatin but Does Not Colocalise with ASYnapsis 1 during Early Meiosis in Bread Wheat

**DOI:** 10.1155/2012/514398

**Published:** 2012-02-06

**Authors:** Kelvin H. P. Khoo, Amanda J. Able, Jason A. Able

**Affiliations:** School of Agriculture, Food & Wine, Waite Research Institute, The University of Adelaide, Waite Campus, PMB1, Glen Osmond, SA, 5064, Australia

## Abstract

Chromosome pairing, synapsis, and DNA recombination are three key processes that occur during early meiosis. A previous study of *Poor Homologous Synapsis 1* (*PHS1*) in maize suggested that PHS1 has a role in coordinating these three processes. Here we report the isolation of wheat (*Triticum aestivum*) *PHS1* (*TaPHS1*), and its expression profile during and after meiosis. While the *Ta*PHS1 protein has sequence similarity to other plant PHS1/PHS1-like proteins, it also possesses a unique region of oligopeptide repeat units. We show that *Ta*PHS1 interacts with both single- and double-stranded DNA *in vitro* and provide evidence of the protein region that imparts the DNA-binding ability. Immunolocalisation data from assays conducted using antisera raised against *Ta*PHS1 show that *Ta*PHS1 associates with chromatin during early meiosis, with the signal persisting beyond chromosome synapsis. Furthermore, *Ta*PHS1 does not appear to colocalise with the asynapsis protein (*Ta*ASY1) suggesting that these proteins are probably independently coordinated. Significantly, the data from the DNA-binding assays and 3-dimensional immunolocalisation of *Ta*PHS1 during early meiosis indicates that *Ta*PHS1 interacts with DNA, a function not previously observed in either the Arabidopsis or maize PHS1 homologues. As such, these results provide new insight into the function of PHS1 during early meiosis in bread wheat.

## 1. Introduction

For the majority of sexually reproducing organisms, meiosis is a cellular process required for gamete formation and is composed of one round of DNA replication, followed by two rounds of chromosome division. During meiosis I, a reductional division event leads to the segregation of homologous chromosome pairs, while an equational division during meiosis II leads to the segregation of the sister chromatids.

 For the successful juxtaposition of homologous chromosomes, three key processes occur during prophase I, namely, pairing, synapsis, and recombination. Previous studies investigating the molecular mechanisms of homologous chromosome pairing have revealed complex interplay between these three tightly linked processes [1–5].

 In allopolyploid organisms such as bread wheat (*Triticum aestivum*), correct alignment and pairing of homologous chromosomes are complicated by the presence of genetically similar genomes, known as homoeologous genomes. Although bread wheat possesses three homoeologous genomes (termed A, B, and D), meiosis proceeds as if the organism is a diploid, in that pairing only occurs between homologous chromosomes from the same genome ([6–9] and references within). This strict pairing interaction between homologous chromosomes has previously been shown to be controlled by *pairing homoeologous* (*Ph*) loci [10, 11]. The most extensively studied of these loci is the *Ph1* locus located on the long arm of chromosome 5B. While the molecular mechanism by which the *Ph1* locus operates is still subject to intensive research, *Ph1* appears to indirectly promote homologous chromosome pairing by suppressing homoeologous chromosome interactions through regulation of the specificity of chromosome interactions at centromeric and telomeric regions [12, 13].

 In *ph1* deletion mutants, the chromatin is prematurely and asynchronously remodelled, leading to abnormal chromosome conformation that results in increased interactions between homoeologous chromosomes [13, 14]. These mutants also display other meiotic defects such as the arrest of synapsis during zygotene and the presence of uncorrected multiple axial element associations, which in wild type are normally corrected prior to entry into pachytene [15, 16]. While the deletion region in the *ph1b* mutant is extensive, the *Ph1* locus has recently been refined to an area that contains, among other genes, seven *Cyclin-dependent kinase*-like (*Cdk*-like) genes [17, 18].

 Our current knowledge of other meiotic genes mostly comes from research on model species such as yeast and Arabidopsis. However, putative homologues of many of these genes have also been identified in the cereals [19]. Some of the early meiotic genes characterised in various plant species include *ASY1* (*ASYnapsis 1*) [20–24], *RAD51* (*RADiation sensitive 51*) [25, 26], and *PHS1 *(*Poor Homologous Synapsis 1*) [27, 28]. In wheat, *ASY1* (*TaASY1*) is involved in chromosome synapsis and promotes homologous chromosome pairing during meiosis I [20, 21]. Interestingly, *Taasy1* knockdown mutants have been reported to display defective chromosome characteristics similar to that of the *ph1b* mutant [20]. In the absence of *Ph1*, the expression of *TaASY1* is approximately 20-fold higher compared to wild type while the localisation of its protein product is also affected. This indicates that *TaASY1* is intimately involved in the *Ph1*-dependent control of chromosome pairing in wheat [9, 20].

*PHS1* was first identified in a *Mutator* transposon-mutagenised maize population, with no known homologues in yeast or other nonplant species [27]. While phenotypic analysis of the *phs1-0* mutant by Pawlowski et al. [27] revealed no vegetative defects, meiosis was disrupted resulting in male and female sterility. Transmission electron microscopy of meiotic spreads from *Zmphs1-0* meiocytes revealed significantly reduced levels of synapsis during zygotene and improper alignment of the chromosomes in the synapsed regions. Although most of the chromosomes were fully synapsed by late pachytene, the chromosomes were synapsed with multiple partners. Coupled together with results of their fluorescent *in situ* hybridisation (FISH), the data indicated that nonhomologous chromosome synapsis was present in the *Zmphs1-0* mutant.

 FISH results from recent work by Ronceret et al. [28] on the Arabidopsis homologue of PHS1 (*At*PHS1) showed that PHS1 appears to function in a similar manner in different species independent of genome size and complexity. Chromosome axis formation and installation of the synaptonemal complex components in both wild type and *phs1* mutant cells of Arabidopsis and maize appeared similar albeit with ZYP1, a transverse filament protein, loading being delayed in some instances [28]. Immunolocalisation in Arabidopsis and maize revealed that PHS1 is located within the cytoplasm during the early stages of meiosis with some foci clustered along the nuclear envelope during zygotene. In some instances, a few foci are present in the nucleus during pachytene. With a reduced number of RAD50 foci observed in the nuclei of the *phs1* mutants, Ronceret and colleagues [28] concluded that PHS1 regulates meiotic recombination and chromosome pairing by regulating the transport of RAD50, a protein which is required during double-strand break processing, into the nucleus.

 Here, we report the extensive analysis of the PHS1 protein in bread wheat, providing evidence that shows *Ta*PHS1 possesses DNA-binding capabilities even though no known DNA-binding domains were identified *in silico*. Our data also show that *PHS1* is upregulated in the *ph1b* bread wheat mutant when compared to wild type, and that *Ta*PHS1 is associated with chromatin and is present on the nucleolar periphery during the early stages of meiosis as indicated through immunolocalisation analysis using an antibody that was raised against the full-length wheat PHS1 protein.

## 2. Materials and Methods

### 2.1. Plant Material

Hexaploid wheat (*Triticum aestivum *L.) cv. Chinese Spring plants and a Chinese Spring mutant lacking the *Ph1* locus (*ph1b*) were grown in a glasshouse with a 16/8 h photoperiod at 23°C. Harvesting and staging of meiotic anthers from both wild type and mutant plants, for quantitative real-time PCR (Q-PCR) and fluorescence immunolocalisation, were conducted as per Boden et al. [20]. Whole meiotic spike tissue was collected for the isolation and amplification of the gDNA and cDNA *Triticum aestivum PHS1* (*TaPHS1*) sequences. 

### 2.2. RNA Isolation and cDNA Synthesis

Collected tissues for RNA isolation were initially ground in liquid nitrogen. Total RNA was extracted using Trizol reagent (Gibco-BRL, Carlsbad, CA, USA) according to the manufacturer's instructions. RNA concentration was determined using a Nanodrop (ND-1000) (Nanodrop, Wilmington, DE, USA). cDNA was synthesised from 2 *μ*g of total RNA using the iScript cDNA synthesis kit (Bio-Rad, Hercules, CA, USA) according to the manufacturer's instructions.

### 2.3. cDNA Amplification and Sequencing of the PHS1 Coding Sequence

Primers (see Table S1 of the supplementary material available online at doi:10.1155/2011/514398) for isolating the *TaPHS1* ORF were designed using the *OsPHS1* sequence (LOC_Os06g27860, MSU Rice Genome Annotation database-http://rice.plantbiology.msu.edu) identified through a TIGR rice expressed sequence tag (EST) BLAST search (accessed 21st October 2008).

 Each PCR contained 100 ng cDNA, 0.2 mM dNTPs, 0.2 *μ*M primers, and 1 U FastStart high-fidelity *Taq* polymerase (Roche Applied Science, Mannheim, Germany) in 25 *μ*L of 1 × high-fidelity buffer supplemented with 1 × GC-RICH solution (Roche). PCR products were cloned into pCR8/GW/TOPO (Invitrogen) for DNA sequencing (15 × coverage). Sequencing PCR and capillary separation were conducted using the same methods as described earlier except that GW1 and GW2 primers were used (see Table S1). Secondary sets of primers were designed on the sequenced products to specifically amplify the *TaPHS1* ORF. Amplification and sequencing were repeated as above. PCR cycling parameters were denaturated at 95°C for 5 min, followed by 35 cycles of 96°C for 30 s, T_m _°C for 30 s, and 72°C for 75 s, with a final elongation step at 72°C for 10 min (see Table S1 for T_m_ of primer sets). The assigned NCBI accession number for *TaPHS1* is GQ851928.

### 2.4. Bioinformatics Analysis

DNA sequence alignments and comparisons were conducted with AlignX and Contig Express (Informax, VNTI Advance, Version 11, Frederick, MC, USA) software programs. VNTI software was also used to predict the molecular weight and pI of the protein. To predict the cellular localisation of *Ta*PHS1, SignalP 3.0 (http://www.cbs.dtu.dk/services/SignalP/) [29] and WoLF PSORT (http://wolfpsort.org/) [30] were used, while detection for conserved domains was performed using the NCBI Conserved Domain Search Tool (http://www.ncbi.nlm.nih.gov/Structure/cdd/wrpsb.cgi), InterPro Scan (http://www.ebi.ac.uk/Tools/InterProScan/), and Pfam 23.0 (http://pfam.janelia.org/).

Amino acid alignments and comparisons of full-length PHS1 sequences (obtained from various BLAST searches using the NCBI, TIGR, and PredictProtein (http://www.predictprotein.org/; [31]) databases) and subsequent construction of the phylogenetic tree (neighbour-joining method) [32] were completed using Molecular Evolutionary Genetics Analysis (MEGA) software (version 4.0) [33]. Default parameters were used except for the following: the pair-wise deletion option was used, the internal branch test bootstrap value was set at 10,000 resamplings, and the model setting was amino acid, Poisson correction with predicted gamma parameters set at 2.0. Accession numbers of the sequences used were, *Ta* [GenBank: GQ851928], *Sb* [TIGR EST assemblies: TA33290_4558], *Zm* [GenBank: NP_001141750]; *Os* [MSU Rice Genome Annotation: LOC_Os06g27860]; *At* [GenBank: NP_172541], *Pt* [UniProtKB: B9HTU7_POPTR]; *Vv* [UniProtKB: A7QY03_VITVI], and *Rc* [UniProtKB: B9SPJ5_RICCO]. To determine whether the level of divergence between *Ta*PHS1 and *Os*PHS1 was significant, a Tajima's Relative Rate Test [34] with the *At*PHS1 sequence as an outgroup was conducted (with one degree of freedom and a significance value of *P* < 0.05). 

### 2.5. Southern Blot Hybridisation

A 371 bp fragment of the *TaPHS1 *gene was used as the template for the synthesis of an *α*-^32^P dCTP labelled probe that was hybridised to a Chinese Spring nullisomic-tetrasomic membrane as per Lloyd et al. [35]. Autoradiography films were developed using an AGFA CP1000 Developer (AGFA, Nunawading, VIC, Australia). For *in silico* mapping experimental procedures refer to supplementary material.

### 2.6. Q-PCR

Q-PCR was conducted in triplicate according to Crismani et al. [36]. Amplification of products was completed using gene-specific Q-PCR primers (see Table S1). The optimal acquisition temperature for *TaPHS1* was 80°C. Normalisation of the expression data was performed against three control genes (*actin*, *GAPdH,* and *cyclophilin*) as per Crismani et al. [36].

### 2.7. Protein Analysis

The *TaPHS1* insert within the pCR8/GW/TOPO vector was cloned into a pDEST17 expression plasmid (Invitrogen) according to the manufacturer's LR clonase protocol. BL-21 A1 *E. coli *were transformed with the pDEST17-*TaPHS1* ORF vector, and protein production was induced with 0.4% L-(+)-arabinose (w/v) (Sigma-Aldrich, St Louis, MO, USA). Production of four partial *Ta*PHS1 peptides corresponding to the four conserved regions identified in this study were also performed as described above using DNA inserts encoding these regions. Protein isolation and purification were performed using nickel-nitrilotriacetic acid (Ni-NTA) beads (Qiagen, Clifton, VIC, Australia) according to the manufacturer's extraction protocols. Sodium dodecyl sulfate polyacrylamide gel electrophoresis (SDS-PAGE) was performed using NuPAGE Novex 4–12% Bis-Tris 7 cm mini-gels (Invitrogen) according to the manufacturer's protocol. Staining and destaining of gels were performed as previously reported [37].

 The identity of the recombinant *Ta*PHS1 protein was confirmed by ion trap liquid chromatography-electrospray ionisation tandem mass spectrometry (LC-MS/MS). Gel slices containing the recombinant *Ta*PHS1 protein were washed with 100 mM ammonium bicarbonate, dried, rehydrated with 100 mM ammonium carbonate, and subjected to in-gel tryptic digestion. LC-MS/MS of the digested peptides was then conducted as reported by March et al. [38].

### 2.8. Polyclonal Antibody Production

Full-length recombinant *Ta*PHS1 protein was dissolved in 1 × PBS (10 *μ*g *μ*L^−1^), added with an equivalent amount of Freund's complete adjuvant (Sigma-Aldrich), and used for primary immunisation of two rats via subcutaneous injection. Three subsequent immunisations were administered in three-week intervals, with Freund's incomplete adjuvant (Sigma-Aldrich) added to the dissolved antigen in 1 × PBS. All immunisation doses contained 200 *μ*g of *Ta*PHS1 antigen. Immune sera was collected 10.5 weeks after the first injection. Specificity of the *Ta*PHS1 antisera was confirmed using western analysis (see supplementary material; Figures S1 and S2).

### 2.9. Competitive DNA-Binding Assay

Recombinant full-length *Ta*PHS1 and the four partial peptides extracted under native conditions were quantified using the Bradford assay [39]. Competitive DNA-binding assays were conducted as described by Pezza et al. [40] with modifications as per Khoo et al. [41]. The DNA-binding abilities of *Ta*PHS1 and its partial peptides were tested with Φ*X*174 circular single-stranded DNA (ssDNA) (virion) (30 *μ*M per nucleotide) (New England Biolabs, Beverly, MA, USA) and Φ*X*174 linear double-stranded DNA (dsDNA) (RFI form *Pst1*-digested) (15 *μ*M per base pair) (New England Biolabs).

### 2.10. Fluorescence Immunolocalisation

Fluorescence immunolocalisation of *Ta*ASY1 and *Ta*PHS1 was performed as per Franklin et al. [42] and Boden et al. [20] with the following changes: anthers were fixed with 2% paraformaldehyde and cells permeabilised for 3 h. For detecting the localisation pattern of *Ta*PHS1, a rat anti-*Ta*PHS1 antisera (1 : 100) and an AlexaFluor 488 conjugated donkey anti-rat antibody (1 : 50; Molecular Probes, Invitrogen) were used. Optical sections (90–120 per nucleus) of meiocytes were collected using a Leica TCS SP5 Spectral Scanning Confocal Microscope (Leica Microsystems, http://www.leica-microsystems.com/) equipped with an oil immersion HCX Plan Apochromat 63 × /1.4 lens, a 405 nm pulsed laser, and an Argon laser using an excitation wavelength of 468 nm. Images were processed using Leica Application Suite Advanced Fluorescence (LAS-AF; version 1.8.2, build 1465, Leica Microsystems) software to generate maximum intensity projections of each nucleus.

## 3. Results

### 3.1. PHS1 Is Highly Similar across Plant Species, and in Wheat It Encodes a Predicted Protein Product with a Unique Oligopeptide Repeat Sequence

**Figure 1:**
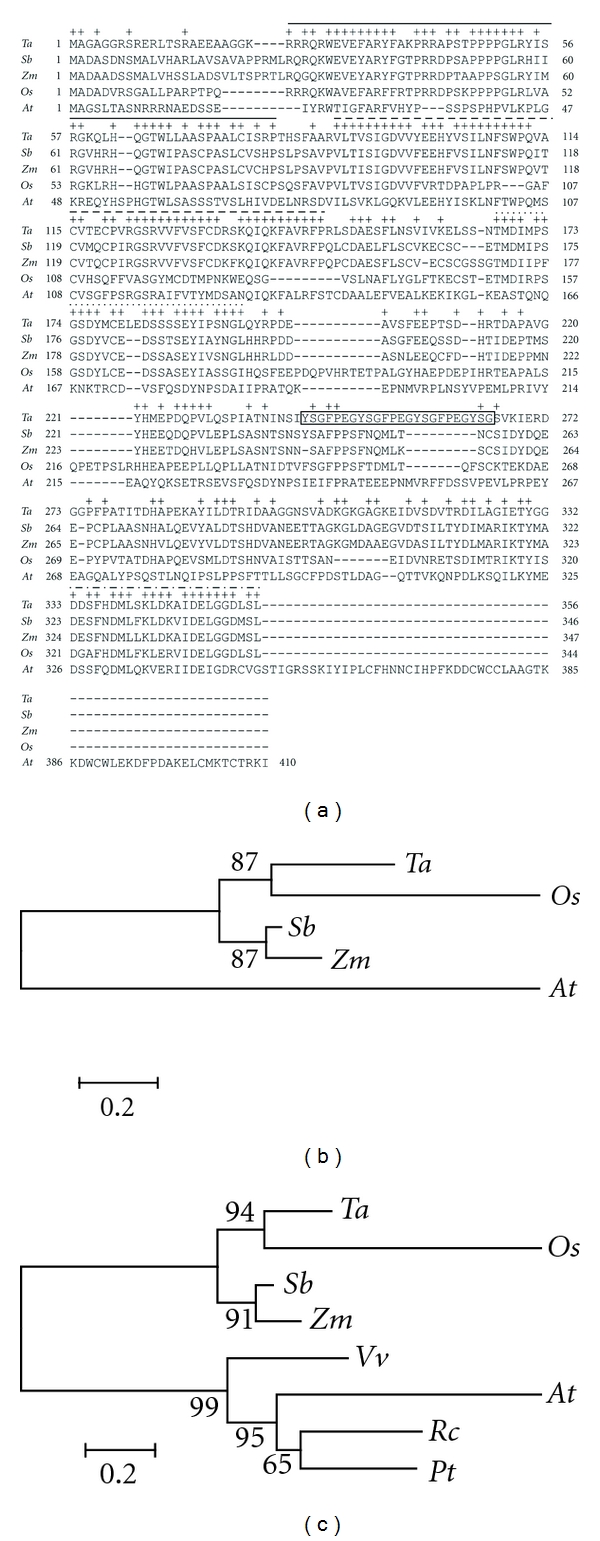
The PHS1 amino acid sequence is well conserved across plant species. (a) Alignment of *Ta*PHS1 with four homologues shows high levels of sequence conservation. Amino acid positions that are conserved across at least three species, using the *Ta*PHS1 amino acid sequence as the reference, are denoted by a ‘‘+” above. Four conserved regions were identified within the PHS1 protein and may represent functional domains: Region 1 (unbroken line), Region 2 (dashed line), Region 3 (dotted line), and Region 4 (dashed-dotted line). Oligopeptide repeat units (denoted by box) unique to *Ta*PHS1 are also highlighted. *Ta*: *Triticum aestivum *PHS1, *Sb*: *Sorghum bicolor *PHS1, *Zm*: *Zea mays *PHS1, *Os*: *Oryza sativa *PHS1, and *At*: *Arabidopsis thaliana *PHS1-like (see Methods for accession numbers). (b) The evolutionary history of *Ta*PHS1 was inferred using the Neighbor-Joining method [32]. (c) Three additional PHS1/PHS1-like sequences were obtained through Hidden Markov Model and MaxHom searches and were also assessed using phylogenetics. *Vv*: *Vitis vinifera *PHS1, *Rc*: *Ricinus communis *PHS1, and *Pt*: *Populus trichocarpa* PHS1. The reliability of the internal branches of the trees (b, c) was assessed with 10,000 bootstrap re-samplings [43], with the confidence probabilities shown next to the branches. The trees are drawn to scale, with branch lengths in the same units as those of the evolutionary distances used to infer the phylogenetic tree. The evolutionary distances were computed using the Poisson correction method [44] and are in the units of the number of amino acid substitutions per site. There were a total of 442 positions in the final datasets. Phylogenetic analyses were conducted in MEGA4 [33].

PCR amplification from whole meiotic spike cDNA using the primers listed in Table S1 (Additional Information File 1) resulted in the isolation of *TaPHS1* which has a 1071 bp ORF. This encodes a 357 amino acid protein with a predicted molecular weight of 38.958 kDa and a pI of 5.23. Despite no nuclear localisation signal (NLS) peptides being detected within the *Ta*PHS1 amino acid sequence using SignalP 3.0, WoLF PSORT analysis predicted that *Ta*PHS1 is most likely to be located within the cell nucleus. Using AlignX, comparative amino acid sequence analysis of full-length annotated PHS1, or PHS1-like proteins obtained through database searches (refer to Methods) showed that *Ta*PHS1 shared relatively high levels of sequence identity with its homologues in other species (*Sorghum bicolor *[*Sb*PHS1]—53.6%, *Zea mays* [*Zm*PHS1]—51.2%, *Oryza sativa* [*Os*PHS1]—41.4%, and *Arabidopsis thaliana* [*At*PHS1-like]—21.5%). We propose that there are four prominent regions within the PHS1 amino acid sequence that are well conserved (Figure 1(a)). While a portion of Region 2 (corresponding to amino acid positions 99–145 of *Ta*PHS1) was previously identified to contain two conserved domains [27], interspecies comparisons made in the current study suggest that this conserved region can now be extended by 11 amino acid residues toward the N-termini of PHS1 proteins in monocot species (Figure 1(a), dashed line). In addition, there is a short region of oligopeptide repeats from position 242 to 265 [YSGFPEGYSGFPEGYSGFPEGYSG] unique to *Ta*PHS1 (Figure 1(a), boxed feature).

To assess the phylogenetic relationships between the five homologues shown in Figure 1(a), a neighbour-joining tree was constructed using the full-length amino acid sequences (Figure 1(b)). As expected, Arabidopsis is the most divergent, while sorghum and maize share a higher degree of similarity with one another. Although wheat and rice fall within the same cluster, the internal branch length difference between the two species suggests that a significant level of sequence divergence has occurred. This significant level of sequence divergence was confirmed by a Tajima's Relative Rate Test (*P* = 0.00015 with one degree of freedom). To determine whether other PHS1 sequences could be identified in the public databases, the more sensitive Hidden Markov Model (HMM) and MaxHom functions of the Predict Protein program were used. Three additional sequences were identified that were similar to *Ta*PHS1 and all from dicot species, namely, poplar (*Populus trichocarpa*) (*E*-value: 7E^−97^), grape (*Vitis vinifera*) (*E*-value: 7E^−91^), and castor oil (*Ricinus communis*) (*E*-value: 2E^−97^). The addition of these three sequences to the phylogenetic analysis shows that they cluster with Arabidopsis, the only other dicot species (Figure 1(c)).

**Figure 2:**
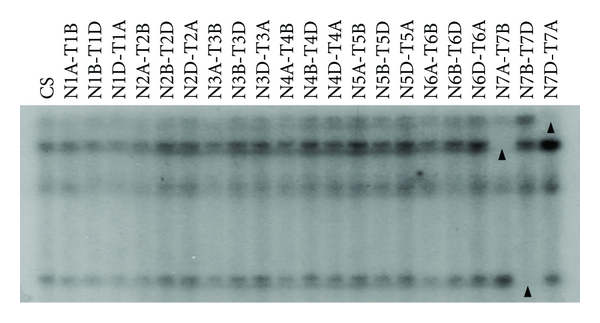
*TaPHS1* is located on chromosome group 7 of wheat. Membranes prepared with DNA from nullisomic (N), tetrasomic (T), and wheat lines of Chinese Spring (CS) were hybridised with a *TaPHS1*-specific probe showing that there is a copy on the A, B, and D genomes of chromosome group 7. Each black arrowhead indicates the absence of a band which represents the presence of a copy of *TaPHS1* in the particular genome for which the plant is nullisomic. The more intense band seen in each lane indicates the presence of the copies of *TaPHS1* in the genome for which the plant is tetrasomically compensated.

 Southern blot analysis showed that *TaPHS1* is located on chromosome group 7, with a copy on each of the three genomes (Figure 2). *In silico,* mapping revealed that *TaPHS1,* is likely to reside on the short arm of this chromosome group (Bin 7AS8-0.45-0.59, data not shown). To determine this, rice genetic markers that are located close to *OsPHS1* (on rice chromosome 6) were used to screen wheat deletion bins. One marker previously bin-mapped to wheat chromosome group 7 [GenBank: BE404111.1] was identified to be approximately 35 kb from *OsPHS1*.

### 3.2. TaPHS1 Interacts with DNA and Is Expressed during Meiosis

**Figure 3:**
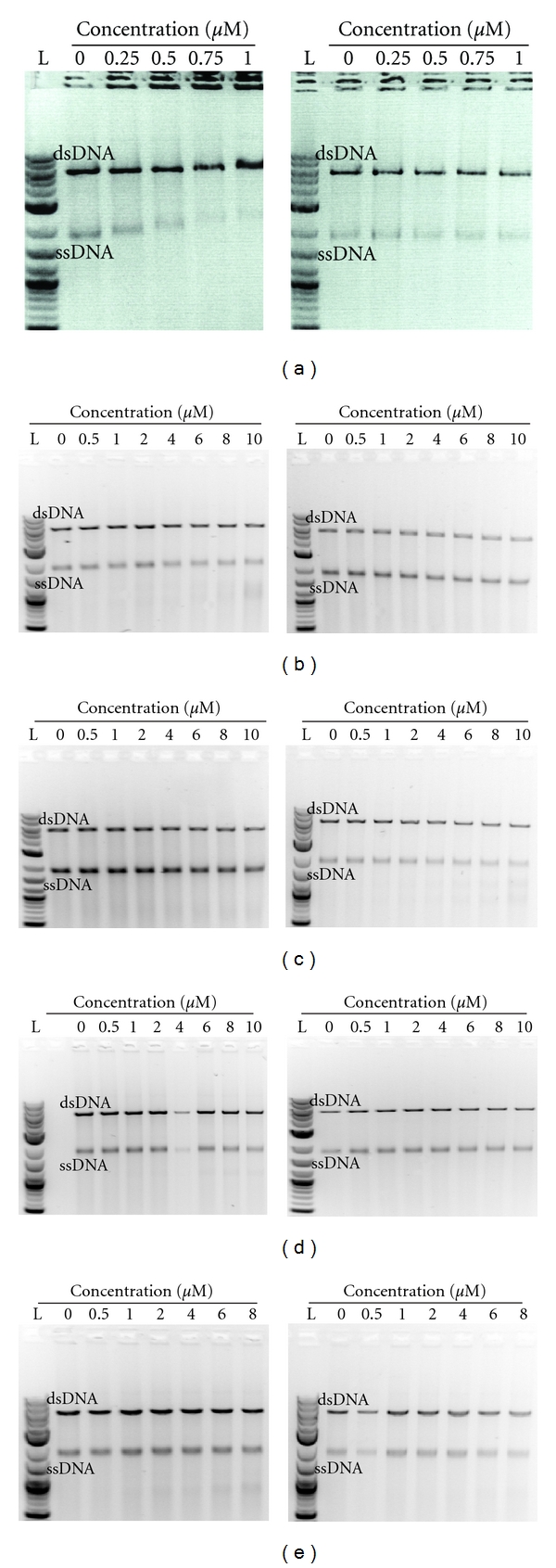
*Ta*PHS1 interacts with DNA *in vitro. *An *E. coli* BL21 cell line containing the pDEST17-*TaPHS1* ORF construct was induced with IPTG (1 mM total concentration) to heterologously produce His-tagged *Ta*PHS1 protein. Total cellular protein was extracted under native conditions, and the His-tagged *Ta*PHS1 was isolated and purified using nickel affinity chromatography. Total cellular protein from the same cell line which was not induced was also extracted and treated identically to be used as the negative control. DNA-binding ability was only observed in assays conducted using the full-length *Ta*PHS1 and the Region 1 peptide, indicating that Region 1 possesses a novel/uncharacterised DNA-binding domain where two S/TPXX putative DNA-binding motifs are located. Using competitive DNA-binding assays with equivalent amounts of single- and double-stranded DNA, *Ta*PHS1 preferentially binds single-stranded DNA (ssDNA). This is evidenced by the increased retardation of the ssDNA species through the gel matrix with increasing concentrations of *Ta*PHS1 that caused more ssDNA to be bounded by *Ta*PHS1. At higher concentrations, *Ta*PHS1 also interacts with double-stranded DNA (dsDNA). (a) Full-length *Ta*PHS1, (b) Region 1 peptide, (c) Region 2 peptide, (d) Region 3 peptide, and (e) Region 4 peptide. Competitive DNA-binding assays performed with the induced samples containing the purified *Ta*PHS1 protein are on the left and noninduced controls are on the right. *μ*M: protein concentration, L: Ladder.

Previously reported homology searches using the maize PHS1 protein revealed low levels of sequence similarity with two families of fungal helicases, possibly indicating that PHS1 may interact with DNA [27]. Indeed, *in silico,* amino acid analysis of *Ta*PHS1 revealed that Region 1 contains two S/TPXX motifs (TPPP: amino acid positions 46 to 49 and SPAA: amino acid positions 71 to 74), which have previously been reported to bind DNA [45]. To determine whether *Ta*PHS1 interacts with DNA, a competitive DNA-binding assay using recombinant *Ta*PHS1 extracted under native conditions was conducted (Figure 3(a)). Interactions occurred with both single-stranded DNA (ssDNA) and double-stranded DNA (dsDNA). Interestingly, *Ta*PHS1 appears to have a higher affinity for ssDNA with retardation of the ssDNA species within the gel matrix being more obvious even when equivalent amounts of ss- and dsDNA are present. Although *Ta*PHS1 also interacts with dsDNA, the retardation of the dsDNA species only occurs at higher concentrations of *Ta*PHS1. Competitive DNA binding assays using partial *Ta*PHS1 peptides corresponding to the four prominent conserved regions identified in this study revealed that only Region 1 possesses DNA-binding capabilities (Figures 3(b)–3(e)) and appears to have a higher affinity for ssDNA compared to dsDNA.

**Figure 4:**
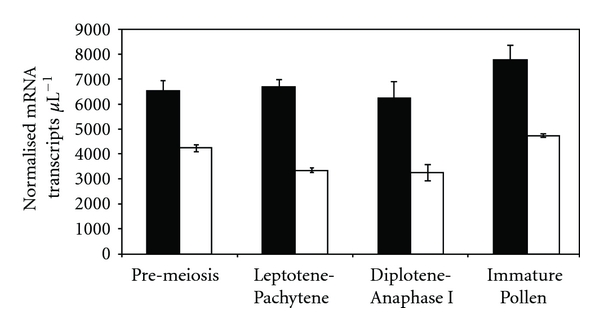
Q-PCR profiling of *TaPHS1* shows that it is expressed during meiosis. While the amount of *TaPHS1* mRNA transcript is low, it has higher levels of expression during premeiosis when compared to the other stages of meiosis examined in Chinese Spring wild type (open bars). In the *ph1b* mutant (black bars), *TaPHS1* is upregulated between 1.5- and 2-fold across the time points analysed. Normalisation of the Q-PCR data was performed against three control genes (*actin*, *GAPdH,* and *cyclophilin*) as per Crismani et al. [36]. Data represent the means ± SE of three replicates. Units on the *y*-axis represent normalised mRNA transcript *μ*L^−1^.

Quantitative real-time PCR (Q-PCR) profiling of *TaPHS1* in wild type Chinese Spring shows that it has low transcript abundance during meiosis (Figure 4). Although *TaPHS1* is expressed in wheat anther tissue throughout all stages of meiosis examined and beyond, statistical analysis suggests that expression is higher during premeiotic interphase and immature pollen. Between the pooled stages of leptotene-pachytene and diplotene-anaphase I, there is no statistically significant difference in *TaPHS1* expression. Given that Boden et al. [20] demonstrated that the *TaASY1* transcript was significantly upregulated in a *ph1b* background when compared to wild type (approximately 20-fold), we also investigated transcription levels of *TaPHS1* in the *ph1b* mutant. While not as dramatic as that reported for *TaASY1* in Boden et al. [20], *TaPHS1* was also upregulated in the *ph1b* mutant when compared to wild type but by between 1.5-fold (premeiosis) and 2-fold (leptotene-pachytene) (Figure 4).

### 3.3. TaPHS1 Is Localised in the Nucleus and Associates with Chromatin during Early Meiosis

**Figure 5:**
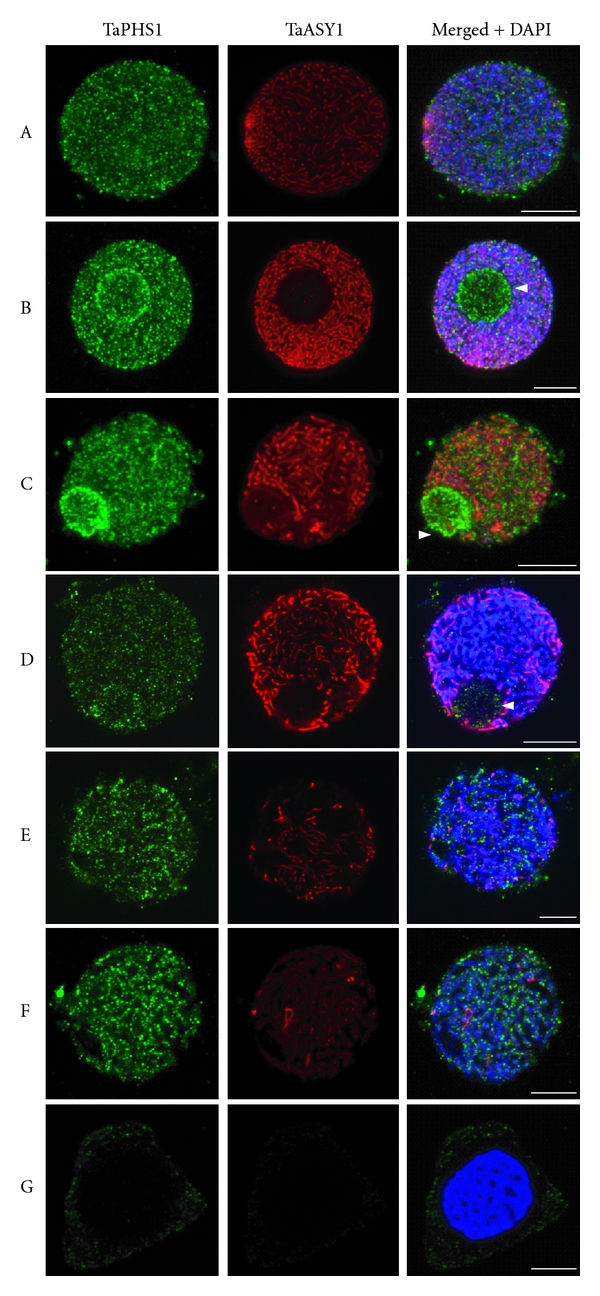
*Ta*PHS1 localisation during early meiosis in wild type bread wheat. (a) Telomere bouquet stage, (b) leptotene, (c) early zygotene, (d) zygotene, (e) late zygotene/pachytene transition, (f) pachytene, (g) diplotene. *Ta*PHS1 (green; left panel) localises to 4′-6-diamidino-2-phenylindole- (DAPI-) stained chromatin (blue) as diffuse tracts and/or punctated foci (as seen by viewing the merged + DAPI image). Middle panels show the *Ta*ASY1 signal (red), while the panels on the right show merged *Ta*PHS1, *Ta*ASY1, and DAPI. Arrowheads (white) represent the nucleolus. Scale bars: 7.5 *μ*m.

3D immunolocalisation of *Ta*PHS1 in wild type wheat meiocytes shows that it associates with chromatin during early meiosis (Figures 5(a)–5(f)). While the signals of both *Ta*PHS1 and *Ta*ASY1 were located within close proximity of each other, the two proteins do not appear to colocalise (e.g., merged panel of Figure 5(f)). In addition to its association with chromatin, the *Ta*PHS1 signal was also observed at the nucleolus (Figures 5(b)–5(e)). This labelling of the nucleolus appears to be on the surface, with a greater signal intensity seen at the nucleolar periphery. This signal appeared to be most intense during early-to-late zygotene/pachytene transition (Figures 5(c)–5(e)). In general, the *Ta*PHS1 signal appeared either as diffuse tracts or punctated foci that follow is the chromatin, unlike the distinct continuous tracts of *Ta*ASY1. The *Ta*PHS1 signal was observed from the telomere bouquet stage and persisted on the chromatin until late pachytene where it faded. Although *Ta*PHS1 was not detected on the chromatin in diplotene cells, detection of a weak signal was still observed in the cytoplasm in what appeared to be randomly distributed foci (Figure 5(g)).

## 4. Discussion

This study has reported the isolation and characterisation of *PHS1* from hexaploid wheat, with the amino acid sequences of *Ta*PHS1 being relatively well-conserved when compared with homologues in other plant species. *In silico,* analysis of the *Ta*PHS1 amino acid sequence suggests that it does not contain any known nuclear localisation signal (NLS) peptide motif. However, predictions using WoLF PSORT show that *Ta*PHS1 fits the profile of a nuclear protein. In addition, the immunolocalisation results (Figure 5) show that *Ta*PHS1 localises to the nuclei of wheat meiocytes *in vivo*. These results together indicate that *Ta*PHS1 and its homologues might possess an uncharacterised NLS motif. Alternatively, the PHS1 protein may be transported into the nucleus by a yet unknown process and/or protein. Given that Region 4, referred to as the CR2 region in Ronceret et al. [28], has been identified as a putative SUMOylation site, there may be no requirement for an NLS motif. Previously, posttranslational modifications such as SUMOylation have been shown to enable transport of proteins from the cytoplasm into the nucleus [46].

 Sequence alignments of *Ta*PHS1 with PHS1 and PHS1-like proteins of four other species obtained from BLAST searches revealed that there is a closer relationship between wheat PHS1 and PHS1 homologues in other cereals than between wheat and Arabidopsis PHS1 (Figures 1(a) and 1(b)). This was not unexpected as cereals are monocots, while Arabidopsis is a dicot. In addition, the Arabidopsis PHS1-like sequence contained an additional 61 amino acid residues on the C-terminal end that were not present in the four monocot species. With the addition of three more dicot PHS1/PHS1-like sequences, individual monocot and dicot branches were still maintained. However, a high level of divergence between the wheat and rice PHS1 sequences was evident (Figure 1(c)) and may indicate that the PHS1 proteins in these two species have evolved to function differently, as is suggested by the presence of the *Ta*PHS1-specific oligopeptide repeat units from position 242 to 265 (Figure 1(a), boxed feature). Another possible explanation for this sequence divergence is that the rice PHS1 sequence, which is putatively annotated as a PHS1 protein, is not a true PHS1 homologue but instead a PHS1-like protein. However, this seems improbable for a number of reasons. Firstly, the rice genome has been sequenced and exhaustive BLAST searches identified *Os*PHS1 as the most significantly similar match to both *Ta*PHS1 and *Zm*PHS1 at the nucleic and amino acid level. Secondly, the *in silico* mapping identified a rice marker on rice chromosome 6 approximately 35 kb away from *OsPHS1 *that is syntenic to a marker that has been bin-mapped to the short arm of wheat chromosome 7. Finally, a significant variance between the amino acid sequences of protein equivalents involved in meiosis has been documented across different organisms, even though their function may be conserved (see [6] and references within).

 The importance of the aforementioned oligopeptide repeat units unique to *Ta*PHS1 remains to be determined. These repeats could be either three tandem hepta-peptide units [YSGFPEG] that span positions 242 to 262 or a series of alternating tripeptide [YSG] and tetrapeptide [FPEG] units that span positions 242 to 265. Comprehensive *in silico* database searches using amino acids 242 to 265 resulted in no significantly similar matches with any repeats reported to date. As single oligopeptide units, the tripeptides, tetrapeptides, and heptapeptides are relatively short and may therefore not form any independent structural units. However, when arranged successively, these oligopeptide units may form a regular repeating structure within *Ta*PHS1, as has previously been reported in other proteins [47]. Based on work previously conducted by Yoder et al. [48], the glutamic acid (E) and glycine (G) residues on the end of the hepta-peptide unit [YSGFPEG] could represent turn residues that link the heptapeptides together, allowing the units to stack on top of each other. This series of oligopeptides may therefore impart a slightly different structure and possibly function for *Ta*PHS1 when compared to the rest of the PHS1/PHS1-like proteins that lack these oligopeptide repeat units. Alternatively, these heptapeptides could have a role similar to the hepta-peptide repeats in the C-terminal domain of the largest subunit of RNA polymerase II, which act as a binding scaffold for protein partners (reviewed by [49]). 

 In contrast to the two conserved regions (termed CR1 and CR2 by Ronceret et al. in [28]) previously described for PHS1 [27], we have shown the presence of four conserved regions (termed Region 1 to 4) within the PHS1 amino acid sequences investigated in this study; with Regions 2 and 4 corresponding with the previously identified CR1 and CR2. Discrepancies in the lengths of the conserved regions identified in the two studies are most likely artefacts of the different alignment algorithms used, in addition to the fact that only full-length annotated transcripts of PHS1 were used in this study. The results of the DNA-binding assays in this study (Figures 3(a) and 3(b)) and the immunolocalisation of *Ta*PHS1 to chromatin (Figure 5) show that *Ta*PHS1 does have DNA-binding capabilities. In the presence of equivalent amounts of ssDNA and dsDNA, *Ta*PHS1 appears to preferentially bind ssDNA *in vitro* but will also bind dsDNA when the protein is present at higher concentrations. Furthermore, we have shown that Region 1 appears to be responsible for the DNA-binding ability (Figure 3(b)). Although the retardation of the DNA is less significant, this can be attributed to the reduced size of the Region 1 partial peptide in comparison to that of the full-length *Ta*PHS1 protein (5.854 kD versus 38.958 kD). Other regions within *Ta*PHS1 may also be required to further enhance the DNA-binding capabilities of Region 1. Bioinformatics analysis of the *Ta*PHS1 protein revealed two S/TPXX DNA-binding motifs previously identified by Suzuki [45] are located within Region 1 (TPPP: amino acid positions 46 to 49; SPAA: amino acid positions 71 to 74), possibly indicating that Region 1 may be a novel DNA interaction domain. Furthermore, our immunolocalisation data suggests that *Ta*PHS1 is closely associated with chromatin (and therefore dsDNA) *in vivo* during early meiosis.

 Although the Q-PCR profiling suggests *TaPHS1* is a low-abundance transcript in the cell, some significant differences were detected during the stages examined, as well as between the wild type Chinese Spring and *ph1b* mutant. While the same general trend of expression is observed in both wild type and the *ph1b* mutant, *TaPHS1* is upregulated in the mutant background by approximately 1.5-fold in pre-meiosis, 2-fold in both leptotene-pachytene and diplotene-anaphase I, and 1.5-fold in immature pollen. This 2-fold increase in expression during early meiosis may suggest that the *Ph1* locus could directly or indirectly affect *TaPHS1*.

 The immunolocalisation results showing *Ta*PHS1 forms diffuse tracts with punctate foci that associate with wheat chromatin are in contrast to the localisation patterns of PHS1 in both maize and Arabidopsis where *Zm*PHS1 and *At*PHS1 form granules within the cytoplasm and not in the nucleus during leptotene and zygotene [28]. In maize, PHS1 was observed to cluster around the nuclear membrane in a small proportion of maize meiocytes during the peak of *Zm*PHS1 accumulation at midzygotene while a few *Zm*PHS1 foci were present in the nucleus during late zygotene. Unlike maize, no such observations were seen with the Arabidopsis PHS1 homologue. This difference in the localisation profile of *Ta*PHS1 compared to its homologues in Arabidopsis and maize may suggest that *Ta*PHS1 instead has a direct role in chromatin interactions.

 The presence of substantial *Ta*PHS1 signal within the nucleoli of early-stage meiocytes may indicate that *Ta*PHS1 is sequestered to the nucleolus either for degradation or storage until required as has been shown for other proteins (reviewed by [50, 51]). Hypothetically, should PHS1 act as a direct physical shuttling protein (as indicated but not favoured by Ronceret and colleagues [28]) that transports specific meiotic proteins into the nucleus; it is likely that it may then be sequestered to the nucleolus for degradation or to undergo further posttranslational modifications to mark it for return to the cytoplasm so that PHS1 molecules can be reused. Although our immunolocalisation data shows that both *Ta*PHS1 and *Ta*ASY1 are loaded and associated with the chromatin at the same time during early meiosis in bread wheat, they do no colocalise with one another. The association of these two proteins with the chromatin appears to be particularly pronounced during late-zygotene to pachytene (Figures 5(e) and 5(f)). Could it be that *Ta*PHS1 is involved in a pachytene check-point mechanism to ensure that only homologous chromosomes have paired and recombined?

Another intriguing result is that the *Ta*PHS1 signal profile appears as faint tracts with punctate foci along regions of the chromatin. Do these punctate foci denote possible recombination sites where *Ta*PHS1 may be loading the recombination machinery? This is plausible as previous reports of recombination proteins including RAD51 [42], RAD50 [28], and MLH3 [52] localise to chromatin as foci as well as the fact that *Ta*PHS1 itself interacts with chromatin. The diffuse tracts of *Ta*PHS1 also suggest a direct role for *Ta*PHS1 in homology searching (in wheat at least) as previously suggested by both Pawlowski and Cande [53] and Ronceret et al. [28]. In the *Zmphs1-0* mutant, RAD50 is not localised to the nucleus preventing the assembly of the MRE11-RAD50-NBS1 complex (collectively known as the MRN complex) thus preventing resection of the DBS, which results in failed recruitment of the RAD51 recombinase protein [27, 28]. RAD51 has previously been shown to be capable of homology searching and promotes homologous chromosome pairing over regions of DNA several kilobases in length [54, 55]. While the reach of the RAD51/DMC1 homology searching nucleoprotein filaments is limited to only a few kilobases, *Ta*PHS1 molecules that form the diffuse tracts may somehow play a direct role in the homology searching process over longer distances of the chromatin. Additional data substantiating this hypothesis was uncovered by Osman and colleagues [56] when they reported that both *At*ZYP1 and *Zm*PHS1 possess regions of peptide sequences that resemble bacterial topoisomerase IV domains. The bacterial topoisomerase IV proteins are members of the topoisomerase type IIA family previously hypothesised to have potential roles in interhomologue chromosome resolution [57]. This fits well into the hypothesis that *Ta*PHS1 may act as a component of the homology searching mechanism in wheat.

## 5. Conclusions

In conclusion, the data presented demonstrates that *Ta*PHS1 has an important and possible novel role during the early stages of wheat meiosis. Data from the DNA-binding assays as well as 3-dimensional immunolocalisation of *Ta*PHS1 during early meiosis in wild type cells indicate that *Ta*PHS1 interacts with DNA, a function not previously observed in the Arabidopsis and maize PHS1 homologues. The localisation signal profile of *Ta*PHS1 may indicate that it is a direct transporter of other meiotic proteins into the nucleus, and that it could have a role in homology searching. While the role(s) of this protein are yet to be fully understood, we are currently in the process of generating *Taphs1* knockdown and *TaPHS1* overexpression mutants to further elucidate the meiotic function in bread wheat.

## Supplementary Material

Supplementary material contains the materials and methods as well as results for the in silico mapping of TaPHS1, western blot assays to confirm the specificity of the anti-TaPHS1 antisera, and a table listing all the primers used in this study.

## References

[1] Armstrong SJ, Franklin FCH, Jones GH (2001). Nucleolus-associated telomere clustering and pairing precede meiotic chromosome synapsis in *Arabidopsis thaliana*. *Journal of Cell Science*.

[2] Chen YK, Leng CH, Olivares H (2004). Heterodimeric complexes of Hop2 and Mnd1 function with Dmc1 to promote meiotic homolog juxtaposition and strand assimilation. *Proceedings of the National Academy of Sciences of the United States of America*.

[3] Higgins JD, Sanchez-Moran E, Armstrong SJ, Jones GH, Franklin FCH (2005). The Arabidopsis synaptonemal complex protein ZYP1 is required for chromosome synapsis and normal fidelity of crossing over. *Genes and Development*.

[4] Kerzendorfer C, Vignard J, Pedrosa-Harand A (2006). The *Arabidopsis thaliana* MND1 homologue plays a key role in meiotic homologous pairing, synapsis and recombination. *Journal of Cell Science*.

[5]  Martínez-Pérez E, Shaw P, Aragon-Alcaide L, Moore G (2003). Chromosomes form into seven groups in hexaploid and tetraploid wheat as a prelude to meiosis. *Plant Journal*.

[6] Able JA, Crismani W, Boden SA (2009). Understanding meiosis and the implications for crop improvement. *Functional Plant Biology*.

[7] Able JA, Langridge P (2006). Wild sex in the grasses. *Trends in Plant Science*.

[8] Able JA, Langridge P, Milligan AS (2007). Capturing diversity in the cereals: many options but little promiscuity. *Trends in Plant Science*.

[9] Moore G, Shaw P (2009). Improving the chances of finding the right partner. *Current Opinion in Genetics and Development*.

[10] Riley R, Chapman V (1958). Genetic control of the cytologically diploid behaviour of hexaploid wheat. *Nature*.

[11] Sears ER (1977). Induced mutant with homoeologous pairing in common wheat. *Canadian Journal of Genetics and Cytology*.

[12] Martínez-Pérez E, Shaw P, Moore G (2001). The *Ph1* locus is needed to ensure specific somatic and meiotic centromere association. *Nature*.

[13] Prieto P, Shaw P, Moore G (2004). Homologue recognition during meiosis is associated with a change in chromatin conformation. *Nature Cell Biology*.

[14] Colas I, Shaw P, Prieto P (2008). Effective chromosome pairing requires chromatin remodeling at the onset of meiosis. *Proceedings of the National Academy of Sciences of the United States of America*.

[15] Holm PB (1988). Chromosome pairing and synaptonemal complex formation in hexaploid wheat, nullisomic for chromosome 5B. *Carlsberg Research Communications*.

[16] Holm PB, Wang XZ (1988). The effect of chromosome 5B on synapsis and chiasma formation in wheat, *Triticum aestivum* cv. Chinese Spring. *Carlsberg Research Communications*.

[17] Al-Kaff N, Knight E, Bertin I (2008). Detailed dissection of the chromosomal region containing the *Ph1* locus in wheat *Triticum aestivum*: with deletion mutants and expression profiling. *Annals of Botany*.

[18] Griffiths S, Sharp R, Foote TN (2006). Molecular characterization of *Ph1* as a major chromosome pairing locus in polyploid wheat. *Nature*.

[19] Bovill WD, Priyanka D, Sanjay K, Able JA (2009). Whole genome approaches to identify early meiotic gene candidates in cereals. *Functional and Integrative Genomics*.

[20] Boden SA, Langridge P, Spangenberg G, Able JA (2009). TaASY1 promotes homologous chromosome interactions and is affected by deletion of *Ph1*. *Plant Journal*.

[21] Boden SA, Shadiac N, Tucker EJ, Langridge P, Able JA (2007). Expression and functional analysis of *Ta*ASY1 during meiosis of bread wheat (*Triticum aestivum*). *BMC Molecular Biology*.

[22] Caryl AP, Armstrong SJ, Jones GH, Franklin FCH (2000). A homologue of the yeast *HOP1* gene is inactivated in the Arabidopsis meiotic mutant *asy1*. *Chromosoma*.

[23] Nonomura KI, Nakano M, Murata K (2004). An insertional mutation in the rice *PAIR2* gene, the ortholog of Arabidopsis *ASY1*, results in a defect in homologous chromosome pairing during meiosis. *Molecular Genetics and Genomics*.

[24] Ross KJ, Fransz P, Armstrong SJ (1997). Cytological characterization of four meiotic mutants of Arabidopsis isolated from T-DNA-transformed lines. *Chromosome Research*.

[25] Bleuyard JY, Gallego ME, Savigny F, White CI (2005). Differing requirements for the Arabidopsis Rad51 paralogs in meiosis and DNA repair. *Plant Journal*.

[26] Li J, Harper LC, Golubovskaya I (2007). Functional analysis of maize RAD51 in meiosis and double-strand break repair. *Genetics*.

[27] Pawlowski WP, Golubovskaya IN, Timofejeva L, Meeley RB, Sheridan WF, Cande WZ (2004). Coordination of Meiotic Recombination, Pairing, and Synapsis by PHS1. *Science*.

[28] Ronceret A, Doutriaux MP, Golubovskaya IN, Pawlowski WP (2009). PHS1 regulates meiotic recombination and homologous chromosome pairing by controlling the transport of RAD50 to the nucleus. *Proceedings of the National Academy of Sciences of the United States of America*.

[29] Bendtsen JD, Nielsen H, von Heijne G, Brunak S (2004). Improved prediction of signal peptides: signalP 3.0. *Journal of Molecular Biology*.

[30] Horton P, Park K-J, Obayashi T (2007). WoLF PSORT: protein localization predictor. *Nucleic Acids Research*.

[31] Rost B, Yachdav G, Liu JF (2004). The PredictProtein server. *Nucleic Acids Research*.

[32] Saitou N, Nei M (1987). The neighbor-joining method—a new method for reconstructing phylogenetic trees. *Molecular biology and evolution*.

[33] Tamura K, Dudley J, Nei M, Kumar S (2007). MEGA4: Molecular Evolutionary Genetics Analysis (MEGA) software version 4.0. *Molecular Biology and Evolution*.

[34] Tajima F (1993). Simple methods for testing the molecular evolutionary clock hypothesis. *Genetics*.

[35] Lloyd AH, Milligan AS, Langridge P, Able JA (2007). *TaMSH7*: a cereal mismatch repair gene that affects fertility in transgenic barley (*Hordeum vulgare L.*). *BMC Plant Biology*.

[36] Crismani W, Baumann U, Sutton T (2006). Microarray expression analysis of meiosis and microsporogenesis in hexaploid bread wheat. *BMC Genomics*.

[37] Wang XX, Li XF, Li YX (2007). A modified Coomassie Brilliant Blue staining method at nanogram sensitivity compatible with proteomic analysis. *Biotechnology Letters*.

[38] March TJ, Able JA, Schultz CJ, Able AJ (2007). A novel late embryogenesis abundant protein and peroxidase associated with black point in barley grains. *Proteomics*.

[39] Bradford MM (1976). A rapid and sensitive method for the quantitation of microgram quantities of protein utilizing the principle of protein dye binding. *Analytical Biochemistry*.

[40] Pezza RJ, Petukhova GV, Ghirlando R, Camerini-Otero RD (2006). Molecular activities of meiosis-specific proteins Hop2, Mnd1, and the Hop2-Mnd1 complex. *Journal of Biological Chemistry*.

[41] Khoo KHP, Jolly HR, Able JA (2008). The *RAD51* gene family in bread wheat is highly conserved across eukaryotes, with *RAD51A* upregulated during early meiosis. *Functional Plant Biology*.

[42] Franklin AE, McElver J, Sunjevaric I, Rothstein R, Bowen B, Zacheus Cande W (1999). Three-dimensional microscopy of the Rad51 recombination protein during meiotic prophase. *Plant Cell*.

[43] Felsenstein J (1985). Confidence-limits on phylogenies—an approach using the bootstrap. *Evolution*.

[44] Zuckerkandl E, Pauling L, Bryson V, Vogel HJ (1965). Evolutionary divergence and convergence in proteins. *Evolving Genes and Proteins*.

[45] Suzuki M (1989). SPXX, a frequent sequence motif in gene regulatory proteins. *Journal of Molecular Biology*.

[46] de Carvalho CE, Colaiácovo MP (2006). SUMO-mediated regulation of synaptonemal complex formation during meiosis. *Genes and Development*.

[47] Katti MV, Sami-Subbu R, Ranjekar PK, Gupta VS (2000). Amino acid repeat patterns in protein sequences: their diversity and structural-functional implications. *Protein Science*.

[48] Yoder MD, Lietzke SE, Jurnak F (1993). Unusual structural features in the parallel *β*-helix in pectate lyases. *Structure*.

[49] Phatnani HP, Greenleaf AL (2006). Phosphorylation and functions of the RNA polymerase II CTD. *Genes and Development*.

[50] Carmo-Fonseca M, Mendes-Soares L, Campos I (2000). To be or not to be in the nucleolus. *Nature Cell Biology*.

[51] Olson MOJ, Dundr M, Szebeni A (2000). The nucleolus: an old factory with unexpected capabilities. *Trends in Cell Biology*.

[52] Jackson N, Sanchez-Moran E, Buckling E, Armstrong SJ, Jones GH, Franklin FCH (2006). Reduced meiotic crossovers and delayed prophase I progression in *At*MLH3-deficient Arabidopsis. *EMBO Journal*.

[53] Pawlowski WP, Cande WZ (2005). Coordinating the events of the meiotic prophase. *Trends in Cell Biology*.

[54] Eggler AL, Inman RB, Cox MM (2002). The Rad51-dependent pairing of long DNA substrates is stabilized by replication protein A. *Journal of Biological Chemistry*.

[55] Nishinaka T, Shinohara A, Ito Y, Yokoyama S, Shibata T (1998). Base pair switching by interconversion of sugar puckers in DNA extended by proteins of RecA-family: a model for homology search in homologous genetic recombination. *Proceedings of the National Academy of Sciences of the United States of America*.

[56] Osman K, Sanchez-Moran E, Higgins JD, Jones GH, Franklin FCH (2006). Chromosome synapsis in Arabidopsis: analysis of the transverse filament protein ZYP1 reveals novel functions for the synaptonemal complex. *Chromosoma*.

[57] von Wettstein D (1984). The synaptonemal complex and genetic segregation. *Symposia of the Society for Experimental Biology*.

