# Supplementary material for: Poor Homologous Synapsis 1 Interacts with Chromatin but Does Not Colocalise with ASYnapsis 1 during Early Meiosis in Bread Wheat
Source: Int J Plant Genomics. 2012 Feb 6;2012:514398. doi: 10.1155/2012/514398 (PMC3303760; doi:10.1155/2012/514398)
Supplement: Supplementary file 1 — Supplementary material contains the materials and methods as well as results for the in silico mapping of TaPHS1, western blot assays to confirm the specificity of the anti-TaPHS1 antisera, and a table listing all the primers used in this study. [file 514398.f1.pdf]

Additional Information File 1

Khoo KHP, Able AJ & Able JA (2011).

Poor homologous synapsis 1 (PHS1) interacts with chromatin but does not co-localise with *Ta*ASY1 during early meiosis in bread wheat.

Materials and Methods

In silico *mapping*

In silico mapping of *TaPHS1* was conducted by first identifying the bacterial artificial chromosome (BAC) [GenBank accession: AP003528] containing the *OsPHS1* genomic sequence [MSU Rice Genome Annotation: LOC_Os06g27860.1] using the Gramene rice database (http://www.gramene.org/Oryza_sativa_japonica/Location/Chromosome?r=6:1-31246789; Gramene Release 29; accessed 20th April 2009). A single-long-sequence (SLS) BLASTN search was performed using the rice BAC sequence as the query. A total of 116 wheat ESTs were identified in the SLS search. Using the rice deletion bin database (http://wheat.pw.usda.gov/wEST/binmaps/), wheat chromosome group 7 shares a high degree of similarity to rice chromosome 6. Each of the 116 identified wheat ESTs was then compared to the available markers on rice chromosome 6 to determine whether the location of *TaPHS1* on wheat chromosome group 7 could be refined.

Western blot

Western blot was performed as per Khoo *et al.* [53]. Membranes were prepared with nickel-affinity purified total protein from induced and non-induced BL21 *E. coli* cell-lines containing the pDEST17-*TaPHS1* ORF plasmid as well as with total cellular protein

extracts from staged meiocytes. The membranes were probed with an alkaline phosphatase-conjugated monoclonal anti-His-tag antibody (1:5000 dilution) (Sigma). Visualisation was performed with the addition of 5-bromo-4-chloro-3′-indoylphosphate *p*-toluidine salt (BCIP) reagent (Bio-Rad, Hercules, CA, USA). The pre-immune sera and terminal bleed post-immunisation sera containing the anti-*Ta*PHS1 antibody were used at a 1:5000 dilution and the antibodies were detected using an alkaline phosphatase-conjugated monoclonal anti-rat IgG (1:10000 dilution) (Sigma).

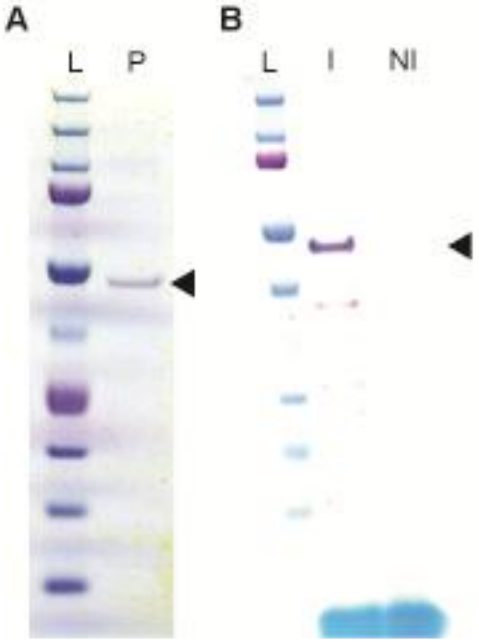

Figure S1 – Western blot of heterologously-expressed *Ta*PHS1. Western blot analysis conducted using an anti-His-tag antibody detected the presence of the His-tagged *Ta*PHS1 that was heterologously-expressed and purified by nickel affinity chromatography under denatured conditions. The most intense band detected by the anti-His-tag antibody was of an identical size to the purified *Ta*PHS1 protein. Three faint bands of lower molecular

weight were also detected but these bands were most likely caused by the detection of degraded His-tagged *Ta*PHS1 protein as no such bands were detected in the non-induced control. (A) SDS-PAGE of His-tagged *Ta*PHS1 protein purified by nickel affinity chromatography, (B) Western blot membrane prepared with nickel affinity purified total protein from induced and non-induced *E. coli* pDEST17-*TaPHS1* ORF cell-lines probed with an anti-His-tag antibody. L = Bio-Rad Precision Plus Protein Ladder; P = Nickel affinity chromatography purified His-tagged *Ta*PHS1; I = Nickel affinity chromatography purified total protein from induced pDEST17-*TaPHS1* cell-line; NI = Nickel affinity chromatography purified total protein from non-induced pDEST17-*TaPHS1* cell-line. Black arrow heads indicate *Ta*PHS1 protein.

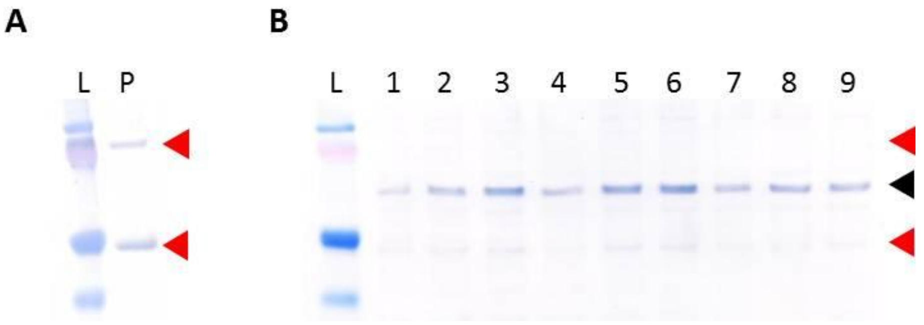

Figure S2 – Detection of plant *Ta*PHS1 protein from staged meiocyte total cellular protein extracts using the *Ta*PHS1 antisera. The specificity of the *Ta*PHS1 antisera against *Ta*PHS1 plant protein was confirmed by using the pre-immune sera and post-immunisation terminal bleed sera. (A) Two non-specific bands (denoted by red arrowheads)

were detected when the pre-immune sera was used to probe total cellular protein extract from a pooled stage of pre-meiotic interphase to anaphase I. (B) Three bands were detected using the post-immunisation terminal bleed sera containing the anti-*Ta*PHS1 antibody. The two non-specific bands (denoted by red arrowheads) previously detected by the pre-immune sera were still present albeit barely visible but the plant *Ta*PHS1 was clearly detected (denoted by the black arrowhead). L = Bio-Rad Precision Plus Protein Ladder; P = pooled total protein extract from meiocytes staged from pre-meiotic interphase to anaphase I; 1 – 3 = three biological replicates of pooled total cellular protein extracts from meiocytes staged from pre-meiotic interphase to pachytene; 4 – 6 = three biological replicates of pooled total cellular protein extracts from meiocytes staged from diplotene to anaphase I; 7-9 = three biological replicates of pooled total cellular protein extracts from meiocytes staged from tetrad to immature pollen.

Table S1 – Detailed list of primers used in this study. Genome walking primers were used for amplification of the 5´ region of *TaPHS1* while open reading frame (ORF)-targeting primers were used to isolate the ORF sequence for protein production. Primers based on the 5´ genomic *TaPHS1* sequence were required to isolate the probe used for the Southern blot hybridisation. Q-PCR primers were used to determine the expression profile of *TaPHS1* in meiotic tissues of both wild-type and *ph1b* mutant plants. Abbreviations: F1, forward 1; R1, reverse 1; Reg1, region 1; Reg2, region 2; Reg3, region 3, Reg4, region 4; QF1, quantitative forward 1; QR1, quantitative reverse 1; SF1, Southern forward 1; SR1, Southern reverse 1.

Primer name	Primer sequence (5´ → 3´)	T_m (°C)
***TaPHS1* gene isolation primers**		
*TaPHS1*_F1	CATTTTCGGCGTCATCGTCGTCG	65
*TaPHS1*_R1	CTACAGTGACAAGTCGCCACCCAGTTCA	
***TaPHS1* gene expression primers (to amplify ORF for protein production)**		
*TaPHS1*_F2	ATGGCGGGCGCCGGC	62
*TaPHS1*_R1	CTACAGTGACAAGTCGCCACCCAGTTCA	
***TaPHS1* peptide primers (to amplify conserved regions for protein production)**		
*TaPHS1*_Reg1_F1	CGGCGGAGGCAGAGGTGG	65
*TaPHS1*_Reg1_R1	GAAGGAGTGGGTGGGGCGAGA	
*TaPHS1*_Reg2_F1	GTCTACGAGGAGCACTATGTATCTATCCTCAAC TT	66
*TaPHS1*_Reg2_R1	AGGGAAACGTACAGCAAACTTCTGGAT	
*TaPHS1*_Reg3_F1	AAGGAACTCTCAAGCAACACCAT	59
*TaPHS1*_Reg3_R1	CGCTTCATCTGGCCTGTATTG	
*TaPHS1*_Reg4_F1	GGCGGGGACGACTCTTTTCAT	62
*TaPHS1*_Reg4_R1	Primer used was *TaPHS1*_R1	
**Plasmid vector sequencing primers**		
T7 (pGEM®-T Easy)	TAATACGACTCACTATAGGG	50
SP6 (pGEM®-T Easy)	ATTTAGGTGACACTATAG	
GW1 (pCR®8/GW/TOPO®)	GTTGCAACAAATTGATGAGCAATGC	50
GW2 (pCR®8/GW/TOPO®)	GTTGCAACAAATTGATGAGCAATTA	
**Quantitative real-time PCR (Q-PCR) primers**		
*TaPHS1*_QF1	CACTCGGATTGATGCTGCTG	55
*TaPHS1*_QR1	TGACAAGTCGCCACCCAGTT	
**Southern blot probe primers**		
*TaPHS1*_SF1	TGAACTGAATTGTGTCGGATGAA	54
*TaPHS1*_SR1	AAAGCTCACGAACACCACCCT	
